# A case report of genetic prion disease with two different *PRNP *variants

**DOI:** 10.1002/mgg3.1134

**Published:** 2020-01-17

**Authors:** Megan Piazza, Thomas W. Prior, Prabhjot S. Khalsa, Brian Appleby

**Affiliations:** ^1^ Center for Human Genetics Laboratory Case Western Reserve University/University Hospitals Cleveland Medical Center Cleveland OH USA; ^2^ Fremont Neurology Medical Associates Fremont CA USA; ^3^ Washington Hospital Healthcare System Fremont CA USA; ^4^ University of California Davis Davis CA USA; ^5^ National Prion Disease Pathology Surveillance Center Case Western Reserve University Cleveland OH USA

**Keywords:** molecular genetics, Prion disease, *PRNP*

## Abstract

**Background:**

Prion diseases are a group of lethal neurodegenerative conditions that occur when the normal, cellular form of the prion protein (PrP^C^) is converted into an abnormal, scrapie, form of the protein (PrP^Sc^). Disease may be caused by genetic, infectious, or sporadic etiologies. The genetic form of prion disease comprises~10%–15% of all cases. Prion disease is typically inherited in an autosomal dominant manner. The low incidence of disease makes it highly unlikely that a patient would have two different pathogenic variants. However, we recently identified a case in which the patient did have two pathogenic *PRNP *variants and presented with an atypical phenotype.

**Methods:**

The patient was evaluated at the Washington Hospital Healthcare System in Fremont, CA. The clinical information for this case report was obtained retrospectively. Variants in the *PRNP* were identified by polymerase chain reaction (PCR) amplification of exon two of the gene followed by bi‐directional sequence analysis. To determine the phase of the identified variants, a restriction enzyme digestion was utilized, followed by sequence analysis of the products. Cerebral spinal fluid (CSF) was analyzed for surrogate markers of prion disease, 14–3–3 and Tau proteins. CSF real‐time quaking‐induced conversion (RT‐QuIC) assays were also performed.

**Results:**

The patient was a compound heterozygote for the well‐characterized c.628G>A (p.Val210Ile) variant and the rare octapeptide deletion of two repeats [c.202_249del48 (p.P68_Q83del)]. Clinically, the patient presented with an early onset demyelinating peripheral neuropathy, followed by later onset cognitive symptoms.

**Conclusion:**

This presentation is reminiscent of prion protein knockout mice whose predominate symptom, due to complete loss of PrP, was late‐onset peripheral neuropathy. To our knowledge this is the first case reported of a patient with prion disease who had two different pathogenic variants in *PRNP*.

## Graphical abstract

Genetic prion disease is typically inherited in an autosomal dominant manner and the low incidence of disease makes it highly unlikely that a patient would inherit two different pathogenic mutations, however, we recently identified a case in which the patient did indeed inherit two pathogenic PRNP variants and presented with an atypical phenotype. The well‐characterized c.628G>A (p.V210I) variant and the rare octapeptide deletion of two repeats [c.202_249del48 (p.P68_Q83del)] was identified in the patient. To our knowledge this is the first case of a patient with prion disease who has two different variants in PRNP.

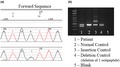

## 1 INTRODUCTION

Prions are proteinaceous infectious particles that lack nucleic acid and are the cause of prion diseases. Prion diseases are a group of lethal neurodegenerative conditions also known as transmissible spongiform encephalopathies. Prion disease occurs when the normal, cellular form of the prion protein (PrP^C^) is converted into an abnormal, scrapie, form of the prion protein (PrP^Sc^) (Prusiner, 1998). The disease‐associated protein (PrP^Sc^) is neurotoxic, which leads to the development of symptoms (Prusiner, 1998). Prion disease may be caused by genetic, infectious, or sporadic etiologies. All forms of prion disease are characterized by the accumulation of abnormal prion protein, spongiosis, gliosis, and neuronal loss (Mastrianni, 2010; Prusiner, 1998). The worldwide yearly incidence of prion disease is ~ 1–2 new cases per million individuals (Holman et al., 2010).

The genetic form of prion disease comprises ~10%–15% of cases (Collinge, 2005; Geschwind, 2015; Mastrianni, 2010). There are more than 60 known variants in the prion protein gene (*PRNP*; OMIM: #176640) that have been linked to inherited human prion diseases (Appleby, Rhoads, Mente, & Cohen, 2018; Prusiner, 1998). These diseases display variable penetrance and are inherited in an autosomal dominant manner (Appleby et al., 2018; Prusiner, 1998). Genetic prion diseases are classified based on the specific variant in *PRNP*, the clinical presentation, and the neuropathologic features. These diseases are separated into three different types. Genetic Creutzfeldt‒Jakob Disease (gCJD) is the most common form of genetic prion disease. The average age of disease onset is between 30 and 55 years and symptoms include rapidly progressive dementia with ataxia and myoclonus. The variant c.598G>A (p.Glu200Lys, aka E200K) is the most common cause of gCJD worldwide. Gerstmann‒Sträussler‒Scheinker (GSS) Syndrome is another type of genetic prion disease. GSS may result from a dozen different variants in the *PRNP *gene. Disease onset for GSS is typically around 50 years and symptoms may include slowly progressive ataxia with late‐onset dementia (Appleby et al., 2018; Geschwind, 2015; Mastrianni, 2010). Fatal Familial Insomnia (FFI) is a genetic form of prion disease caused by the *PRNP *variant c.532G>A (p.Asp178Asn, aka p.D178N) in cis with the c.385A (p.129M) polymorphism (Appleby et al., 2018). Disease onset is typically between 40 and 50 years and symptoms include severe progressive insomnia, followed by dysautonomia with later onset motor and cognitive symptoms (Mastrianni, 2010). Disease presentation may vary for any of the genetic forms of prion disease, even within individual families.

### 1.1 Genetic prion disease

Many variants in the *PRNP *have been identified in patients with genetic prion disease. The types of variants seen may be divided into two main groups. One group consists of missense and nonsense variants in the *PRNP *gene. These variants result in an amino‐acid modification in the C terminal domain which alters the function of the protein or creates a premature stop codon, leading to a truncated protein (Beck et al., 2010). The second group of variants consists of copy number variants, deletions or insertions, of the number of octapeptide repeats in the N‐terminal domain of the prion protein (Beck et al., 2010). Alteration of the number of octapeptide repeats may hinder the function of the protein.

The majority of pathogenic *PRNP *variants are missense variants (Geschwind, 2015). To date, there are approximately 50 known pathogenic missense variants in the *PRNP *(The Human Gene Mutation Database). The first *PRNP* mutation to be identified in patients with GSS was the c.305C>T (p.Pro102Leu) variant (Prusiner, 1998). This variant has since been identified in many GSS families throughout the world. In addition to this early identified variant, other variants have been readily found in patients with gCJD and include c.532G>A (p.D178N) in cis with the c.385A>G (p.Met129Val, aka p.M129V) polymorphism, c.598G>A (p.E200K), and c.628G>A (p.Val210Ile, aka p.V210I) (Beck et al., 2010).

The most common polymorphic variant of the *PRNP *is the c.385A>G (p.Met128Val, aka p.M129V) variant. This alteration plays an important and complex role in the risk of prion disease and the phenotype observed in the patient. The common allele seen in the majority of the population is the c.385A (p.129M) variant. Approximately 43% of the population is homozygous for the c.385A (p.129M) variant and ~ 48% is heterozygous for the c.385A>G p.M129V variant (ExAC database). Only ~ 9% of the general population is homozygous for the c.385G (p.129V) variant (ExAC database). In some instances the c.385A>G (p.M129V) variant influences the disease phenotype. This may be reflected in the age of onset of the symptoms, the course of the disease, or the specific symptoms seen in the patient. For example, the variant c.532G>A (p.D178N) is heavily influenced by the c.385A>G (p.M129V) variant. When the c.532G>A (p.D178N) variant is in cis with the c.385A (p.129M) allele, the disease phenotype is FFI (Mastrianni, 2010). When the c.532G>A (p.D178N) variant is in cis with the c.385G (p.129V) allele the presentation is more similar to that of sporadic CJD (Mastrianni, 2010). Additional studies are still needed to determine if other variants are influenced by the c.385A>G (p.M129V) polymorphism.

The human prion protein contains an octapeptide repeat region (octarepeats; R1‐R2‐R2‐R3‐R4) that consists of five repeats. The five repeats are made up of one 24–27 bp segment that encodes a nonapeptide (R1 = PQGGGGWGQ) and four repeats that encode octapeptide coding sequences (R2, R3, R4 = PHGGGWGQ)] (Li, Qing, Yan, & Kong, 2011). Variation in the repeat sequence may lead to prion disease (Li et al., 2011; Mastrianni, 2010). The clinical and pathological phenotypes associated with these pathogenic copy number variants are heterogeneous and heavily influenced by the number of octapeptide repeats. It is hypothesized that the octapeptide repeat insertion leads to disease by rendering the corresponding mutant PrP protein more prone to adopting a prion‐associated conformation (Li et al., 2011). While large octapeptide repeat insertions lead to prion disease, small octapeptide repeat insertions have not been found to segregate in families with disease. However, it may be difficult to distinguish rare polymorphisms from incompletely penetrant variants. There have been rare cases reported with one octapeptide repeat insertion in patients with neurological disease (Laplanche, et al., 1995; Pietrini et al., 2003). However, one and three octapeptide repeat insertions have also been identified in control populations (Beck et al., 2010). When repeat insertions of three or less are identified in patients the phenotype is most consistent with gCJD. The presence of four or more repeats is associated with a GSS phenotype (Geschwind, 2015; Mastrianni, 2010; Takada et al., 2016).

In addition to the pathogenic insertions of the octapeptide repeat region, octapeptide repeat deletions have also been described as a cause of prion disease. Two‐octapeptide repeat deletions have been reported as pathogenic variants in patients with symptoms of disease (Beck et al., 2001; Capellari et al., 2002). However, deletion of a single 24‐bp octapeptide repeat region has not been found to associate with disease and is considered a normal variant (Beck et al., 2010; Mastrianni, 2010).

Genetic prion diseases are inherited in an autosomal dominant manner and patients with two different variants (compound heterozygotes) are rare. The compound heterozygous cases that have been reported are patients with an established *PRNP *variant and a single octapeptide repeat insertion on the other chromosome. However, the one octapeptide repeat is common and considered a polymorphism (Beck et al., 2010). The low incidence of prion disease makes it highly unlikely that a patient would have two different pathogenic variants. In rare cases de novo variants in the *PRNP* have been reported (Brown & Mastrianni, 2010). In this report we have identified a case in which the patient had two pathogenic *PRNP *variants and presented with an atypical phenotype.

## 2 MATERIALS AND METHODS

### 2.1 Ethical compliance

All procedures were performed under the protocols approved by the Institutional Review Board at Case Western Reserve University and University Hospitals, Cleveland, OH, USA. All patient data and samples were coded and handled according to NIH guidelines to protect patient identities.

### 2.2 Clinical Evaluation

The patient was evaluated at the Washington Hospital Healthcare System in Fremont, CA. Cerebral spinal fluid and postmortem brain biopsy specimens (fixed and frozen tissue) were sent to the National Prion Disease Pathology Surveillance Center and the Center for Human Genetics Laboratory in Cleveland, OH for protein and genetic analyses. The clinical information for this report was obtained in a retrospective manner.

### 2.3 Sanger Sequencing

Variants in the *PRNP* were identified by polymerase chain reaction (PCR) amplification of exon two of the gene followed by bi‐directional sequence analysis. Three primers were utilized for amplification of exon two: PrionF CCATTGCTATGCACTCATTCA, PrionR AGAAAGAGTGAGACACCACCA, and INT5 TGCATGTTTTCACGATAGTAACGG. Four primers were used for sequencing the region of interest: PrionF CCATTGCTATGCACTCATTCA, PrionR AGAAAGAGTGAGACACCACCA, INT5 TGCATGTTTTCACGATAGTAACGG, and INT3 CCGTTACTATCGTGAAAACATG. To determine the phase of the identified variants, a Bsa AI restriction enzyme digestion was utilized, followed by sequence analysis of the products. The reference sequence used for the *PRNP* was NM_000311.3.

### 2.4 Protein Analyses

Surrogate markers for prion disease, 14–3–3 and tau protein, were measured by western blot and enzyme linked immunosorbent assays (ELISA) respectively, as previously described (Hamlin et al., 2012). Real‐time quaking‐induced conversion (RT‐QuIC) assays were performed as previously described (Foutz et al., 2017; Orrú et al., 2015).

## 3 CASE REPORT

### 3.1 Clinical Information

The patient was a 62‐year‐old male who was in a normal state of health until he developed symptoms of dizziness and disequilibrium. Other symptoms included unsteadiness on his feet and numbness in both distal lower and upper extremities. Over the course of 2 weeks his presenting symptoms significantly progressed and he developed an intermittent diplopia. Two months later, the patient began to experience progressive dementia, personality changes, psychosis, progressive ataxia, alien limb, and myoclonus.

An extensive neurological workup was performed. An electromyogram (EMG) and nerve conduction study (NCS) revealed a demyelinating peripheral neuropathy with axonal features consistent with Guillain‒Barre Syndrome. The patient had two magnetic resonance imaging (MRI) analyses that were both suggestive of CJD. However, an electroencephalogram (EEG) study was not suggestive of CJD. A sample of CSF was sent to the National Prion Disease Pathology Surveillance Center (NPDPSC) in Cleveland, OH for protein analyses. The sample had a tau level of 5,950, was positive for 14–3–3, and was positive via RT‐QuIC analysis. Brain tissue samples were also analyzed by the NPDPSC and the neuropathological presentation of the patient's brain was consistent with MM(MV)1 (Appleby et al., 2018). In addition, the PrP^Sc^ electrophoretic profile was type 1 prion protein (typing based on Parchi et al., 1999). Due to the positive neurological workup and the protein analyses, the patient was diagnosed with CJD.

The patient ultimately succumbed to the disease 3 months after the onset of the initial symptoms. Postmortem genetic analysis on brain biopsy specimen was done at the Center for Human Genetics Laboratory in Cleveland, OH. Genetic analysis identified two variants in the *PRNP* gene. The well‐characterized c.628G>A (p.Val210Ile aka p.V210I) variant and the rare octapeptide deletion of two repeats [c.202_249del48 (p.P68_Q83del)] were identified in the patient's sample. There was no family history of neurodegenerative disease or other neurologic diseases, including CJD. To our knowledge, no genetic testing for these specific *PRNP* variants was performed on family members. Therefore, it was unknown if the variants identified were inherited or de novo. The patient previously worked as an electrician and no additional information was known about the patient's life events, including possible exposure history.

The patient's presentation of a demyelinating peripheral neuropathy was atypical for genetic CJD. However, approximately 28.4% of sporadic CJD cases have symptoms suggestive of peripheral neuropathy and some *PRNP* variants have been seen in patients with autonomic neuropathy (Baiardi et al., 2019; Bommarito et al., 2018). The unique genetic component of the patient's disease, two variants identified in the *PRNP *gene, may provide some insight into the unusual clinical presentation.

### 3.2 Genetic Information

**Figure 1:**
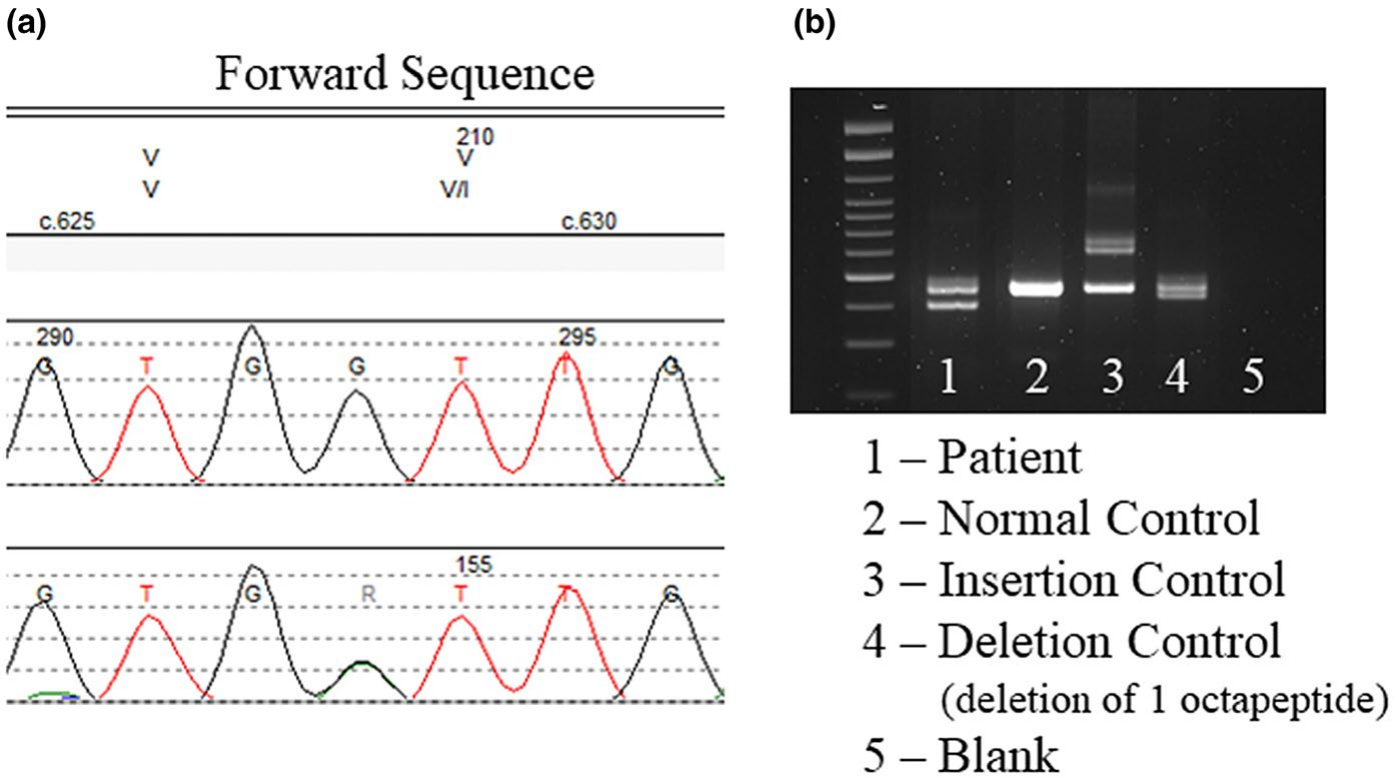
Genetic analyses of a patient identified two different variants in *PRNP.* (a) Sanger sequencing analysis identified the c.628G>A (p.V210I) variant. Top: Amino acid sequence, Middle: Reference sequence, Bottom: Patient sequence. (b) PCR and gel electrophoresis showing the deletion in the patient's sample (lane 1) as compared to the controls (see figure for key). Sequence analysis determined that the deletion was two repeats [c.202_249del48 (p.P68_Q83del)]. The reference sequence used for *PRNP* sequencing was NM_000311.3

The well‐characterized c.628G>A (p.V210I) variant and the rare octapeptide deletion of two repeats [c.202_249del48 (p.P68_Q83del)] were identified in the patient **(**Figure 1 **)**. The patient in this report was missing the third and fourth repeats (R2 and R3) of the normal octapeptide repeat sequence (48 bp deletion, R2 R3). Additional molecular analyses determined that these two variants were located *in trans*. The patient was also homozygous for methionine at the polymorphic codon 129 [c.385A (p.129M)]. Based on the patient's phenotype, reports on *PRNP *knock‐out mouse models, and previous reports suggesting the 48‐bp octapeptide repeat deletion is pathogenic, we conclude that the atypical clinical phenotype is the result of two different variants in *PRNP*.

Missense variants in *PRNP *have been reported in patients with genetic prion disease. The c.628G>A (p.V210I) variant has been reported in many patients with prion disease (Bagyinszky, Giau, Youn, An, & Kim, 2018; Biljan et al.., 2011; Furukawa., Kitamoto, Hashiguchi, & Tateishi, 1996; Imbriani et al., 2016; Mouillet‐Richard et al., 1999; Nozaki et al., 2010; Pocchiari et al., 1993; Ripoll et al., 1993; Shyu, Hsu, Kao, & Tsao, 1996; Tajima, Satoh, Mito, & Kitamoto, 2014). Mouse models inoculated with brain extracts from patients with the p.V210I variant developed symptoms of prion disease ~200 days postinoculation (Mastrianni et al., 2001). Patients with this variant have been shown to experience symptoms similar to sporadic CJD. These symptoms include: early onset vertigo, personality change, and headache, and later onset dementia and ataxia (Schelzke, Kretzschmar, & Zerr, 2012). The mean age of disease onset is ~50 years and the mean duration of disease is ~4 months (Schelzke et al., 2012). In addition, patients typically lack a positive family history of prion disease. This may be due to either reduced penetrance or a higher de novo rate as compared to other documented *PRNP *variants (Mastrianni et al., 2001; Schelzke et al., 2012).

The human prion protein gene has an octapeptide repeat region that consists of five repeats of 24–27 bp sequences. Alterations to this repeat region may lead to the development of prion disease. Insertions or deletions in this region are hypothesized to occur by replication slippage or recombination between the repeats (Beck et al., 2001; Li et al., 2011). Pathogenic duplications of the *PRNP* octapeptide repeats range from 1 to 12 and the clinical and pathological phenotypes are heterogeneous (Kumar et al., 2011; Li et al., 2011). Deletions of the octapeptide region of the *PRNP *are less well characterized. Deletions of a single octapeptide repeat are found to be a normal polymorphism in the population and are not considered pathogenic of prion disease (Beck et al., 2001). However, there are two reports of patients having deletions consisting of two octarepeats, suggesting the 48‐base pair deletion is pathogenic (Beck et al., 2001; Capellari et al., 2002).

The first case was reported by Beck et al in 2001 and described an 86‐year‐old female who experienced progressive forgetfulness, confusion, tremors, and a gait abnormality (Beck et al., 2001). There was no family history of neurodegenerative disease. The patient passed 23 months after the onset of the disease as a result of the symptoms. No additional studies were performed postmortem. A diagnosis of probable CJD was cited. Genetic analysis determined that the patient was homozygous for methionine at the polymorphic codon 129 [c.385A (p.129M)] and had a deletion of two‐octapeptide repeats [deletion of the third and fourth repeats (R2 and R3)] (Beck et al., 2001). No other single base pair alterations were detected. The two‐octapeptide repeat deletion was not identified in a study that analyzed > 3,000 control individuals (Beck et al., 2001).

The second case report was described by Capellari et al in 2002 and consisted of a 62‐year‐old male who sustained a head injury due to a motor vehicle accident. After the injury he developed symptoms of dizziness, personality changes, and behavioral abnormalities (Capellari et al., 2002). He then developed myoclonus and rapidly progressive dementia. The family history was positive only for dementia in the patient's mother. The patient succumbed to the disease 18 months after disease onset. The diagnosis of CJD was given following an autopsy examination and analysis of the prion protein. Genetic analysis determined that the patient was homozygous for methionine at the polymorphic codon 129 [c.385A (p.129M)] and had a deletion of two‐octapeptide repeats [deletion of the second and third repeats (R2 and R2)] (Capellari et al., 2002). No other single base pair alterations were detected.

These two case reports describe the same 48‐bp deletion that is seen in our patient and all three cases display the clinical and pathologic features of CJD. In addition, the two‐octapeptide repeat deletion was not identified in an analysis of the general population. Taken together, these three cases support the hypothesis that the two‐octapeptide deletion is a pathogenic variant.

This case report varies slightly from the other two reports, as two different variants in the *PRNP *were identified. This patient was compound heterozygous for the c.628G>A (p.V210I) variant and the octapeptide deletion of two repeats [c.202_249del48 (p.P68_Q83del)]. Additional analyses determined that these two variants were *in trans*. To our knowledge, this is the first report of a patient with genetic prion disease with two different pathogenic variants in the *PRNP *gene. Table 1 outlines the clinical differences seen in patients with the p.Val210Ile variant, two‐octapeptide repeat deletions, and the patient presented in this report with both alterations.

**Table 1:** Description of typical cases with the c.628G>A (p.Val210Ile) variant and two‐octapeptide repeat deletions based on previously reported cases. Description of the atypical case with both variants in *PRNP*. The reference sequence used for *PRNP* sequencing was NM_000311.3

Variant	Disease Type	Mean Age of Disease Onset	Mean Duration of Disease	Symptoms	Reference
c.628G>A (p.Val210Ile)	Typical	~50 years	~4 months	Early onset: vertigo, personality change, and headache Late onset: dementia and ataxia	Ripoll et al. (1993), Pocchiari et al (1993), Furukawa et al. (1996), Shyu et al. (1996), Mouillet‐Richard et al. (1999), Nozaki et al. (2010), Biljan et al. (2011), Schelzke et al. (2012), Tajima et al. (2014), Imbriani et al. (2016), Bagyinszky et al. (2018)
Deletion of Two‐Octapeptide Repeats	Typical^a^	~62–86 years	~18–23 months	Early onset: Forgetfulness, confusion, dizziness, personality change, and tremors Late onset: Myoclonus and rapidly progressive dementia	Beck et al. (2001), Capellari et al. (2002)
c.628G>A (p.Val210Ile) + Deletion of Two‐Octapeptide Repeat	Atypical^b^	~62 years	~3 months	Early onset: Demyelinating peripheral neuropathy Late onset: Personality change, psychosis, ataxia, myoclonus, and progressive dementia	This report

a Based on two reports

b Based on one report

## 4 DISCUSSION

In this report we present a genetic case of prion disease with an unusual peripheral neuropathy phenotype and two variants in the *PRNP *gene. This is an interesting presentation as CJD in itself is a rare disease and homozygosity for variants in the *PRNP *represents an even rarer occurrence. Genetic CJD with two homozygous variants of c.598G>A (p.E200K) have been described, but compound heterozygosity for two different *PRNP* variants has not, to our knowledge, been described before (Simon et al., 2000).

A study was conducted to determine the phenotypic differences in patients with genetic CJD that were either heterozygous or homozygous for the c.598G>A (p.E200K) variant. The study group utilized was one of the original founder populations of the c.598G>A (p.E200K) variant, Libyan Jews living in Israel. The study analyzed 70 patients and included 5 that were homozygous for the variant and 65 that were heterozygous for the variant (Simon et al., 2000). Overall, it was determined that the disease features and the duration of disease between the two groups were not statistically different. However, the age of disease onset was significantly (*p* = .03) younger in the homozygous group (50.4 ± 6.2 years) as compared to the heterozygous group (59.1 ± 9.0 years; Simon et al., 2000). The small sample size was likely a factor for the lack of variation in the phenotype between the two groups. Also of note, of the five patients homozygous for the c.598G>A (p.E200K) variant, all but one had detectable levels of PrP. Additional analyses of genetic CJD cases with homozygous *PRNP *variants would be necessary to fully characterize the difference between heterozygous and homozygous *PRNP* variants.

Mouse models have been created to elucidate the pathogenicity of the prion protein. Early *Prnp *knockout models displayed a range of symptoms, likely due to genetic confounders due to the *Prnp *targeting vector and the breeding schemes utilized to generate the models (Nuvolone et al., 2016). Despite the variation in the models, the development of a late‐onset peripheral neuropathy was the one symptom that was present in five out of the seven models created (Nuvolone et al., 2016; Wulf, Senatore, & Aguzzi, 2017). This common finding in the various models confirmed that the prion protein plays a role in peripheral myelin maintenance and complete knockout of the protein interferes with this function (Nuvolone et al., 2016; Wulf et al., 2017).

The patient presented in this case report developed a demyelinating peripheral neuropathy. This may suggest an abnormality of the prion protein in the patient, which altered the maintenance of the peripheral myelin (Nuvolone et al., 2016). It is important to note that the clinical phenotype differed between the patient and the mouse models in that the demyelinating peripheral neuropathy was the presenting symptom in the patient. Peripheral neuropathy was an insidious late‐onset symptom in the mouse models. Based on the protein analyses of the patient's sample it was determined that the two different variants did not lead to a complete loss of prion protein function, but could have altered protein function enough to lead to the phenotype seen. Peripheral neuropathy is not an uncommon clinical manifestation of sporadic CJD and is dependent on molecular subtype (Baiardi et al., 2019). Less is known about the prevalence of peripheral neuropathy in genetic forms of prion diseases, specifically in the type of disease due to the variants seen in this patient. Of note, the patient's clinical phenotype did vary from the phenotypes previously described in the other two cases with the c.628G>A (p.V210I) and 2‐OPRD variants.

Additional research, via animal models or analyses of patients, is needed to fully elucidate the effects of multiple pathogenic variants. Until this may be accomplished, we may speculate on the effects of homozygous *PRNP *variants. Homozygous variants in *PRNP*, as compared to compound heterozygous loss, may be creating more robust dosage effects and thus affecting symptom development. This effect may lead to an increased amount of prion protein or may alter the function of the protein. This in turn may contribute to the early age of disease onset seen in the patients homozygous for the c.598G>A (p.E200K) variants or to the unique phenotype such as seen with the patient in this case report (Simon et al., 2000).

In conclusion, we have identified a unique case of genetic CJD that had two different *PRNP* variants. The patient was compound heterozygous for the well‐characterized c.628G>A (p.V210I) variant and the more rare octapeptide deletion of two repeats [c.202_249del48 (p.P68_Q83del)]. He was also homozygous for methionine at the polymorphic codon 129 c.385A (p.129M). The initial symptoms included an early onset peripheral neuropathy, followed by later‐onset cognitive symptoms. The presence of two different variants in the *PRNP *may have created an altered PrP which contributed to the unique clinical phenotype of the patient.

## CONFLICT OF INTEREST

The authors have no conflict of interest to disclose.
